# Biometals as Potential Predictors of the Neurodegenerative Decline in Alzheimer’s Disease.

**DOI:** 10.7759/cureus.5573

**Published:** 2019-09-05

**Authors:** Liseth K Lavado, Michelle H Zhang, Karan Patel, Sohim Khan, Urvish K Patel

**Affiliations:** 1 Nursing, Rutgers School of Nursing, Newark, USA; 2 Psychological & Brain Sciences and Biology, Johns Hopkins University, Baltimore, USA; 3 Neuroscience, Johns Hopkins University, Baltimore, USA; 4 Public Health, Icahn School of Medicine at Mount Sinai, New York, USA; 5 Neurology and Public Health, Icahn School of Medicine at Mount Sinai, New York, USA

**Keywords:** alzheimer’s disease, mild cognitive impairment, amyloid-𝛽, biometals, copper, calcium, iron, neurodegenerative disease, zinc

## Abstract

Alzheimer’s Disease (AD) is a debilitating neurodegenerative disease that is diagnosed by gradual memory loss and certain cognitive impairments involving attention, reasoning, and language. Most of the research on Alzheimer’s disease focuses on the correlation of its neuropathological changes in the neurofibrillary tangles caused by hyper-phosphorylated tau protein and β-amyloid plaques with respect to cognitive impairment. Its pathology, however, remains incompletely understood. Currently, research has demonstrated that environmental factors such as biometals play a crucial role in exacerbating AD progression. The present review examines the role of metals in AD progression and how metal dyshomeostasis attributes to AD pathogenesis.

It was found that certain metals possess both beneficial and harmful properties in terms of AD progression. Depending upon the concentration of the metal of interest, copper, zinc, iron, and selenium have general beneficial properties. However, when present in excess, they can lead to oxidative stress and hyperphosphorylation of tau protein, amongst other harmful effects, while calcium and magnesium were seen to have beneficial effects by regulating biometal uptake.

In this review, we have provided evidential studies that focus on the involvement of certain metals in antioxidant pathways leading to the formation of reactive species indicative of neurodegeneration.

## Introduction and background

Alzheimer’s disease (AD) is an irreversible brain disorder that affects over 6 million individuals. Ten percent of the current population over the age of sixty-five sufferers from AD, and this percentage doubles for every five years past this age. As a result, half the population over the age of eighty suffers from AD. Although research funding for Alzheimer’s is higher than it has ever been, there is still no effective therapeutic agent to this disease. AD originates in the medial temporal lobe and follows the perforant path from the entorhinal cortex to the dentate gyrus, before moving to the frontal lobe toward the posterior cingulate cortex. It is a progressive disease and has an intermediate form known as Mild Cognitive Impairment (MCI) before it is clinically diagnosed as Alzheimer’s Disease [1].

In the most common form of MCI, patients express concern over the fact that they are beginning to lose memory, performing significantly worse on memory tasks compared to age-matched healthy controls. Some patients with MCI go on to develop full-blown AD, with demonstrable psychological, behavioral and cognitive deficits. In terms of the symptoms corresponding to AD, patients face psychological deficits including hallucinations or paranoia. The most prominent behavioral and cognitive deficits in patients with AD are emotional agitation and severe mental and cognitive declines well beyond those of age-matched controls [1]. 

The primary neuropathological signatures of Alzheimer’s Disease (AD) are amyloid plaques and intraneuronal neurofibrillary tangles, which are formed from the aggregation of insoluble hyper-phosphorylated tau protein and amyloid-𝛽 (Aβ). Aβ is a proteolytic fragment of a precursor of a transmembrane protein, while tau is a microtubule-associated protein. Over recent years, it has been shown that, in AD pathogenesis, Aβ causes the conversion of wildtype tau to a hyperphosphorylated-version. This results in a positive feedback loop where hyper-phosphorylated tau increases the neurotoxicity of Aβ, which in turn causes an increase in hyper-phosphorylated tau [2]. 

Recent research on AD has shed light on the possible effects of environmental factors which can contribute to the progression of AD. Specifically, research has shown that certain biometals are able to favor the maturation of Aβ protein aggregation, leading to further toxicity [3]. These biometals include copper, zinc, magnesium, and calcium, among others. These distinct metals are important for a variety of reasons, from the spatial co-localization of copper, zinc, and iron, to the regulating effects of calcium and magnesium, to the cross-linked effects of copper, selenium, and iron in AD mediation [3-5]. 

Many of these metals can be seen to be beneficial in moderate, physiological concentrations/amounts, but when observed to be in excess, they can prove harmful and exacerbatory to AD pathology. Majority of the benefits that these biometals provide are seen in their ability to either support cellular antioxidants or act as antioxidants themselves [3,6,7]. It is important to identify these beneficial effects of biometals to better understand the necessity of homeostasis, as a deficiency may result in impaired cellular processes and increased oxidative stress [3]. Aside from these benefits, though, certain metals are detrimental in excess concentrations, contributing to Aβ secretion and hyper-phosphorylation of tau; for example, in high concentrations, zinc and copper will promote these processes, thus increasing oxidative stress. Often, these processes are paired with metal reductions and the formation of toxic free radicals. It is widely known that AD pathology is greatly related to the effects of Aβ aggregation--neuroinflammation--and those of hyper-phosphorylated tau--neurofibrillary tangles. Hence, it is in the best interests of AD patients, to determine amounts of these metals in the body and establish ways of preventing dyshomeostasis. 

This review paper examines current research on the function of biometals on the onset and progression of AD. Particularly, we examine and discusses the role of copper, zinc, magnesium, calcium, iron and selenium in AD pathology and provide information on how changes in metal concentrations can exacerbate disease progression in elderly individuals. We aimed to present a detailed overview of both the harmful effects of excess high concentrations of certain biometals and the beneficial mediating roles of other metals in regulated amounts. 

## Review

Metals of interest

The biometals that have been implicated in AD progression are copper, zinc, magnesium, calcium, iron and selenium, primarily because of the role these metals play in regulating the neuronal synaptic activity. In particular, research has shown that Cu2+, Zn2+, and Fe2+ are found in the core and rims of senile plaques in AD patients and co-localize with Aβ [4]. Therefore, altering the baseline levels of these metals can interfere with homeostasis within synapses and stimulate the production of free radicals, which in turn can trigger neurodegeneration [3]. Particularly, copper, iron, zinc, magnesium and calcium play a role in inducing Aβ protein aggregation and oxidative stress [7]. For instance, iron acts as a catalyst for Aβ protein aggregation by forming reactive oxygen species (ROS) [3], whereas the other metals are involved in other pathways. Although not much research is available on selenium, limited research has shown that selenium can also induce free radical formation and oxidative stress [3]. 

Role of metals in AD

Copper and Zinc

Copper is an essential micronutrient and a redox-active metal that has been associated in AD pathogenesis. It is needed by Superoxidant Dismutase 1 (SOD1), which is a vital cellular antioxidant [7]. It has also been found that the interaction of Cu2+ and Aβ forms hydrogen peroxide (H_2_O_2_), as a byproduct, which further contributes to the formation of hydroxyl radicals related to AD pathogenesis [6]. While zinc provides structural support to SOD1, an excess concentration of Zn2+ contributes to the hyper-phosphorylation of tau, a cytosolic protein involved in microtubule assembly and stability [6,7]. An overabundance of copper and zinc can also be detrimental by promoting the production of reactive oxygen species (ROS), which can further damage proteins, DNA, and lipids [7]. For instance, in mice model studies, it was found that molecules designed to chelate Zn2+ and Cu2+ from Aβ aggregates increased Aβ solubilization, subsequently decreasing Aβ deposits [6]. Additionally, research has found that super-stoichiometric levels of these metals result in insoluble and amorphous aggregates, which aggregate into oligomeric precursors rather than organized fibrils [6]. This eventually leads to the formation of tau-paired helical filaments (PHF), which ultimately gather to form neurofibrillary tangles (NFT). 

Additionally, zinc and copper are implicated in AD through complications associated with metallothioneins. Metallothioneins belong to a family of ubiquitous proteins and are capable of binding metals and serving as antioxidants [6]. In particular, neuronal metallothionein 3 (MT3) is involved in the transport and homeostasis of Zn2+ and Cu2+, which helps to reduce Aβ aggregation and increase soluble amyloid precursor proteins (sAPPα) [6]. sAPPα helps to promote neuronal survival and neurite outgrowth [6]. MT3, in particular, interacts with Aβ to abolish Cu2+ mediated aggregation [6]. As a result, a defect in the MT3 pathway can lead to the increased aggregation that is seen in AD patients. 

Magnesium and Calcium

Magnesium and calcium play a role in AD through their relationship with AβPP and presenilins. Additionally, zinc and copper are implicated in the pathway of presenilins. Presenilins are evolutionarily conserved proteins, which serve as the γ-secretase multi-protein complex catalytic subunit and act as novel regulators of cellular zinc and copper uptake [5]. The γ- secretase multi-protein complex is responsible for cleaving AβPP; however, mutations resulting in overexpression of presenilin, particularly in the genes encoding presenilins PS1 and PS2, are crucial indicators of the majority of AD cases [5]. Mutations in presenilins result in the downregulation of Ca2+ channels and Ca2+ dependent mitochondrial transport proteins that strengthen the relationship between presenilin and Ca2+ homeostasis. This downregulation is evident in familial forms of AD [6]. Research has suggested that both Ca2+ and Mg2+ stabilize γ-secretase and enhance its activity [6], which contributes to a decrease in Aβ. 

Iron

Research has shown that Aβ aggregation in the brain leads to inflammation and oxidative stress resulting in increased iron deposition [6]. In a positive feedback cycle, increased iron deposition causes more oxidative stress, which then contributes to early Aβ deposition [8]. As a result, Aβ has been found to alter redox-inactive ferric iron (Fe3+), which is generally present as a reserve form of iron (ferrihydrite), to redox-active ferrous iron (Fe2+). Redox-inactive ferrous iron acts as the catalyst of Fenton reactions that produce toxic free radicals, which subsequently drive neuroinflammation [9]. For instance, it has been shown that elderly individuals who exhibit mild cognitive impairment (MCI) exhibit an increased iron deposition in the cortex and cerebellum [4]. Additional research has shown that there is a relationship between AβPP and iron homeostasis. Particularly, AβPP contains a non-canonical iron response element (IRE) in the 5’ uncoded region of mRNA, which allows the translation of mRNA to be placed directly under iron regulatory proteins (IRPs), specifically IRP1 [10]. This suggests that an increase in intracellular levels of iron leads to increased amounts of AβPP [10]. As a result, an excess of iron contributes to Aβ production and leads to annular protofibrils, which slows the formation of ordered cross-β fibrils, leading to more disordered and toxic aggregates [6]. Additionally, AβPP plays a crucial role in iron homeostasis as it contains a sequence within the protein that allows it to interact with ferroportin and improve iron export [11]. 

Another key to maintaining proper iron levels is hepcidin, a regulatory hormone that is synthesized in the liver and allows for a decreased release of iron [12]. Hepcidin regulates iron via ferroportin, an iron export protein located on the cell surface of macrophages, enterocytes, and hepatocytes, and leads to its internalization and degradation in order to prevent cellular export of iron. The interaction between AβPP with ferroportin promotes iron export and its ferroxidase activity [6]. Additionally, just as zinc promotes hyper-phosphorylation in the tau protein, Fe3+ induces aggregation by binding to tau; likewise, a reduction in Fe2+ could reverse the aggregation of tau [6]. This suggests that iron dyshomeostasis could contribute to AD neuroinflammatory pathology because an excess of iron can lead to oxidative stress and perpetuate tau hyper-phosphorylation [6]. 

Research has also suggested that iron expression of iron metabolism-associated proteins such as divalent metal transporter 1 (DMT1)--a proton-coupled metal ion transport--and ferroportin 1 (FPN1), which is responsible for iron absorption in the intestines due to its role of exporter of iron, could affect the iron load in the brain [13]. 

Selenium

The function of selenium is twofold. First, varying the concentrations of selenium can help mediate AD. For instance, high levels of selenium increase the formation of free radicals due to its ability to oxidize endogenous sulfhydryl groups [3]. However, selenium also acts as an antioxidant. The effects of selenium as an antioxidant are particularly seen through a pathway that involves glutathione peroxidase (GSH-Px). A deficiency in selenium can, therefore, interfere with GSH-Px and lead to oxidative stress [3].

Findings from prior studies

Effects of Copper and Zinc in mediating AD

In terms of the role of presenilins in mediating AD, a study on Drosophilia melangogaster showed that Presenilin knockdown was related to an elevated susceptibility to paraquat, a potent inducer of superoxide [5]. The inability of Presenilin to regulate copper and zinc uptake leads to an increase in the concentrations of these metals and contributes to metal dyshomeostasis, which further leads to the oxidative stress seen in AD patients [5].

Effects of Calcium in mediating AD

Hyper-phosphorylation of tau proteins, one of the pathological dysfunctions of AD, results when microtubule formation is inhibited, which leads to neurofibrillary tangles and instability of the cytoskeletal system, causing double helix fibers. These double helix fibers are the main structures in AD and eventually elicit neuronal death [14]. Calcium/calmodulin-dependent protein kinase IV (CAMK4) is a serine/threonine kinase that phosphorylates transcription factors that control memory consolidation. The principal role of CAMK4 is to phosphorylate microtubule-associated protein 2 and tau protein. CAMK4 is activated by calmodulin (CALM) binding to Ca2+ when intracellular Ca2+ levels increase. This favors calcium-calmodulin complex formation, which then activates calcium/calmodulin-dependent protein kinase kinase 1 (CAMKK1) through phosphorylation. CAMKK1 subsequently activates CAMK4 by phosphorylation [14].

Genistein (GS), a 4′,5,73 hydroxy isoflavone compound from soybean and other plant species, is a promising compound that has shown neuroprotective effects in treating and preventing AD by reducing the deposition of Aβ, resisting toxicity and inflammatory damage, regulating calcium levels, and reducing apoptosis in the hippocampus [15]. Furthermore, GS was seen to improve learning and memory in AD model rats [14]. 

In a study done to evaluate the neuroprotective role of GS, researchers utilized the Morris water maze test to measure the memory consolidation ability of 80 female Sprague Dawley rats aged 10 months. The study tracked the escape latency-time taken to find and climb onto the platform-of four groups of rats: the sham group, the AD group, the GS group, and estradiol valerate (EV) group as a positive control group. It revealed that the AD rats’ escape latency increased, meaning that they took more time to find and climb unto the platform, and the number of times they were able to successfully cross the original platform location after removal of the platform was significantly reduced in comparison to the sham group. When compared to the AD group though, the GS and EV groups had significantly reduced escape latencies and increased number of original platform crossings, indicating that GS improved learning and memory in AD model rats [14]. Moreover, it was also found that the hippocampal neuronal cells of the GS group were closely packed and uniform in color compared to those of AD group, which were disordered, and some even died as characterized by cell shrinkage. On the other hand, the sham group cells were strongly packed, displayed intact colored nuclei and hyperchromatic nucleoli [14]. The EV group hippocampal neurons increased in number and were rarely stained, in contrast to the AD group, whose cells exhibited dark red staining after lysis. These results indicate that GS exhibits a neuroprotective effect. Further, p-CAMK4, CALM and CAMKK1 levels were highest in the hippocampus in the AD group compared to the sham, GS and EV groups [14], indicating that GS can reduce these levels through calcium regulation, playing an important role in the mediation of AD.

Iron association in neuroinflammatory AD pathology

In a study performed to examine iron-associated neuroinflammatory AD pathology, researchers imaged 5 control human hippocampal and 5 AD brain specimens using 7T MRI at ultra-high resolution. The aim was to quantify the location of iron with respect to microglia and Aβ. Microglia activated through the Fenton reaction are responsible for imitating an inflammatory response in AD patients. It was hypothesized that iron accumulation would be found within activated microglia instead of Aβ, which would indicate the inflammatory components of AD [15]. The results of this study identified many minor MR hypointensities within the subiculum of the hippocampus. This was best explained by a combination of activated microglia (p= 0.025) and microscopic iron, as opposed to the involvement of tau and amyloid, which were less prevalent [15]. These results indicate that the presence of iron, specifically Fe2+, influences microglia activation and subsequently causes an inflammatory reaction that leads to neurodegeneration. 

Likewise, in another study, researchers assessed whether there were differences in the serum level of hepcidin and other related factors (serum iron, serum ferritin, % saturation and total iron-binding capacity (TIBC)) as potential markers for AD and MCI. The study sample consisted of 52 AD patients (pure AD was confirmed in 19 patients via autopsy), 9 MCI patients, and 24 control patients. Frozen serum samples of the subjects were collected and analyzed using enzyme-linked immunosorbent assay (ELISA) to measure hepcidin serum levels, ferritin, and TIBC. It was found that the iron-related serum measurements were higher in AD patients in comparison to controls and that the group differences in serum ferritin were statistically significant (p= 0.025) [12]. The study found high serum ferritin levels to be observed in both MCI patients and patients with early-stage AD-stages in which there are relatively low serum iron levels [12]. This suggests that inflammation could be the cause of the observed increase in serum ferritin as ferritin is made up of two subunits characterized by their molecular weight, H and L, with H subunits increasing during inflammation and oxidative stress [12]. This increase of H subunits can disrupt the H/L ratio and lead to H-subunit derived ferroxidase activity and higher levels of unliganded iron, which have the potential for cellular damage [12]. 

Cross-linked effects of Copper, Selenium and Iron in mediating AD

The previously presented idea that there is an increase in serum iron levels in AD patients was confirmed by another study conducted on 32 AD patients comparing the concentration of copper, selenium, and iron in elderly AD patients to the serum concentration of healthy control subjects [3,12]. The researchers found that patients with a Clinical Dementia Rating (CDR) level of CDR-2 and CDR-3 exhibited high levels of iron (p=(0.009, 0.017) and copper (p=0.013, 0.0010), with CDR-3 patients exhibiting lower selenium levels than the healthy controls (p=0.008) [3]. They concluded that the increase in copper levels was due to ceruloplasmin enzyme expression. Ceruloplasmin enzyme binds to copper and decreases its unrestricted concentration and so increased expression of this enzyme would account for the increased copper levels. Additionally, the high levels of copper was found to be directly proportional to the peroxide levels, which increases the concentrations of free radicals and subsequently increase oxidative stress in patients [3]. Lastly, the researchers mention that the accumulation of high levels of iron in brain regions such as the hippocampus, basal ganglia, cerebral cortex, and neurofibrillary tangles, or around senile plaques could lead to neurodegeneration [3], which supports the small MR hypointensities found within the subiculum of the hippocampus of the aforementioned study [15]. Table 1 summarizes the effect of biometals in AD.

**Table 1:** Biometals and their effects. AβPP: Amyloid-β Protein Precursor; MT3: Metallothionein 3; Cu^2+^: Cupper;  Zn^2+^: Zinc; Mg^2+^: Magnesium; Ca^2+^: Calcium; Fe^2+^: Ferritin

Metal	Iron-Binding Protein	Effect
Cu^2+^	Amyloid-β (Aβ) peptide	Harmful: Interaction between Cu^2+^and Aβ is correlated with a metal reduction from Cu(II) -> Cu(I) along with the formation of H_2_O_2_, leading to hydroxyl free radicals [6]. Aβ promotes oxidative stress in the presence of redox metals, copper, and iron [6].
Cu^2+^	Metallothionein 3 (MT3)	Harmful: Defects in MT3 pathway lead to increased aggregation of Aβ as MT3 is involved with the homeostasis of Zn^2+^ and Cu^2+^ by increasing soluble amyloid precursor proteins (sAPPα) [6].
Zn^2+^	Amyloid-β (Aβ) peptide	Beneficial [at low levels]: Competes with iron/copper to bind to Aβ to prevent Cu-Aβ induced formation of H_2_O_2_ and toxic free radicals [6]. Harmful [in excess]: Oxidants releasing excess zinc can trigger neuronal death that may be related to the toxic effect of Aβ [6] Studies found that high concentrations of zinc-binding to Aβ force Aβ to participate over a wide range of pH [16], and preserve the highly ordered conformation of Aβ, producing toxic, fibrillar Aβ aggregates [6].
Zn^2+^	Tau Protein	Harmful [in excess]: The pathological concentration of Zn^2+^ dramatically accelerated the abnormal aggregation of full-length human Tau [in Sh-SY5Y cells] [17], in addition to enhancing Tau aggregation in neuronal cells and Tau aggregation-induced apoptosis and toxicity. Contributes to hyperphosphorylation of tau [6], and lead to the formation of tau-paired helical filaments (PHF) that gather into neurofibrillary tangles (NFT).
Mg^2+^	Amyloid-β Protein Precursor (AβPP)	Beneficial: Decreased intracellular Ma^2+^ levels impair cell viability; magnesium modulates AβPP processing as follows [18]. High Mg^2+^ promotes non-amyloidogenic AβPP cleavage through α-secretase while demoting amyloidogenic processing by β-secretase [18], which is typically associated with the formation of amyloid plaque [19]. Lowering Mg^2+^ concentration sees the opposite, detrimental effects [18].
Ca^2+^	AβPP & Presenilins	Beneficial: Both Ca^2+^ and Mg^2+^ stabilize γ-secretase (which cleaves AβPP), enhancing its activity and decreasing Aβ secretion [6]. Mutations in presenilins downregulate Ca^2+^ channels and Ca^2+^ dependent mitochondrial transport proteins, which is seen in familial forms of AD [6]
Fe^2+^	Amyloid-β peptide	Harmful: Increased iron deposition leads to oxidative stress, contributing to early Aβ deposition [8]. Excess of iron leads to annular protofibrils, slowing formation of ordered cross-β fibrils and resulting in disordered, toxic aggregates [6]. In short, excess iron is linked to neuron loss in AD brain [20], iron enhances the toxicity of Aβ in cultured neural cells [21]. Iron in reduced Fe^2+^ state accelerates cell damage through toxic hydroxyl radical formation catalyzation [10].
Fe^2+^	Tau protein	Harmful: Fe^3+^ induces aggregation through tau-binding Reduction of Fe^2+ ^can reverse this aggregation Iron dyshomeostasis can contribute to AD neuroinflammation by causing oxidative stress and tau hyperphosphorylation [similar to zinc] [6].
Fe^2+^	Divalent metal transport 1 (DMT1) and Ferroportion 1 (FPN1)	Harmful: Expression of these iron metabolism-associated proteins could affect iron load in the brain negatively [13].
Selenium	Endogenous sulfhydryl groups	Harmful [in excess]: In high concentrations, Se can oxidize endogenous sulfhydryl groups, increasing free radical formation [22]. Beneficial [in moderation]: Acts as an antioxidant component Deficiency results in glutathione peroxidase (GSH-Px) impairment, resulting in oxidative stress [23].

Table 2 mentions brief information from previous studies showing the evidence of ion-binding proteins in the progression of AD.

 

**Table 2:** The studies showing the evidence of ion-binding proteins in the progression of Alzheimer’s Disease Cu^2+^: Cupper;  Zn^2+^: Zinc; Mg^2+^: Magnesium; Ca^2+^: Calcium; Fe^2+^: Ferritin

Problem	Metal	Effect	Model
ProSAP/Shank scaffold proteins	Zn^2+^ Ca^2+^	Zn sequestering by Aβ (Amyloid-β) decreases Shank1 and ProSAP27Shank3 protein levels and promotes synapse loss by disruption of Homer1b and Shank1 scaffold [24,25]. Homers 2 and 3 interact with Amyloid-β Protein Precursor (APP), inhibiting APP processing and consequently reducing AB secretion [19,20,26,27].	Primary hippocampal cells, human brain tissue, and Cos7 cells HEK293 cells, C57/black6 mouse model
Ferritin	Fe^2+^	Increases protein levels. Present within and around amyloid plaques and neurofibrillary tangles [28-30].	Human brain tissue
S100B	Ca^2+^ Zn^2+^ Ca^2+^	Increased expression of S100B contributes to overexpressing β-APP in diffuse amyloid deposits [31]. S100B interacts with tau resulting in the inhibition of tau phosphorylation via Ca/calmodulin-dependent kinase II [32,33].	Primary neuron cells from fetal rats Bovine S100B, SH-SYSY cells
S100A9	Ca^2+^	Increases protein levels. Present near amyloid plaques. Interacts with Aβ in vitro and forms linear and annular aggregates. Knockout of the S100A9 gene reduces neuropathology due to reduced Aβ and APP C-terminal levels [34-37].	Human brain tissues, Tg2576 mice model, SH-SYSY cells, and S100A9 recombinant protein.
S100A7	Ca^2+^	Expression of exogenous S100A7 inhibits AB production and promotes a-secretase activity [38].	Primary corticohippocampal cells

## Conclusions

The latest research on AD suggests that the progression of AD is influenced by the presence of biometals. That is why it is important to study how the dyshomeostasis and interaction of certain metals can induce AD as there is presently no cure for AD. Recent studies indicate that certain metals, especially in high concentrations, can induce the formation of Aβ aggregates and reactive oxygen species leading to oxidative stress. The data in the research indicates that the presence of excess iron in particular influences the progression of AD as it undergoes the Fenton reaction and forms free radicals. Likewise, other biometals such as magnesium and calcium interfere with the cleavage of AβPP and presenilin function, which further causes the formation of Aβ aggregates and oxidative stress. Similarly, copper has been shown to mediate the progression of AD because of the role it plays in the presenilin pathway. The current research provides evidence on the effects of metals in AD and suggests that treatment options for AD should focus on balancing the dyshomeostasis of the metals involved. 
